# Overexpression of MTA1 inhibits the metastatic ability of ZR-75-30 cells in vitro by promoting MTA2 degradation

**DOI:** 10.1186/s12964-019-0318-6

**Published:** 2019-01-14

**Authors:** Long Zhang, Qi Wang, Yuzhen Zhou, Qianwen Ouyang, Weixing Dai, Jianfeng Chen, Peipei Ding, Ling Li, Xin Zhang, Wei Zhang, Xinyue Lv, Luying Li, Pingzhao Zhang, Guoxiang Cai, Weiguo Hu

**Affiliations:** 1 Fudan University Shanghai Cancer Center and Institutes of Biomedical Sciences, Collaborative Innovation Center of Cancer Medicine, Shanghai Medical College, Fudan University, 270 Dong’an Road, Shanghai, 200032 China; 2 Department of Immunology, Shanghai Medical College, Fudan University, 130 Dong’an Road, Shanghai, 200032 China; 3 Cancer Institute, Fudan University Shanghai Cancer Center, Fudan University, 270 Dong’an Road, Shanghai, 200032 China; 4 Department of Colorectal Surgery, Fudan University Shanghai Cancer Center, 270 Dong’an Road, Shanghai, 200032 China; 5 Department of Oncology, Shanghai Medical College, Fudan University, 130 Dong’an Road, Shanghai, 200032 China; 6 Department of Breast Surgery, The Third Hospital of Nanchang, China Jiangxi Province Key Laboratory for Breast Diseases, 2 South Xiangshan Road, Nanchang, 330009 Jiangxi China

**Keywords:** MTA1, MTA2, Neutrophil elastase, Elafin, Breast cancer metastasis

## Abstract

**Background:**

As the first member of the metastasis-associated protein (MTA) family, MTA1 and another MTA family member, MTA2, have both been reported to promote breast cancer progression and metastasis. However, the difference and relationship between MTA1 and MTA2 have not been fully elucidated.

**Methods:**

Transwell assays were used to assess the roles of MTA1 and MTA2 in the metastasis of ZR-75-30 luminal B breast cancer cells in vitro. Immunoblotting and qRT-PCR were used to evaluate the effect of MTA1 overexpression on MTA2. Proteases that cleave MTA2 were predicted using an online web server. The role of neutrophil elastase (NE) in MTA1 overexpression-induced MTA2 downregulation was confirmed by specific inhibitor treatment, knockdown, overexpression and immunocytochemistry, and NE cleavage sites in MTA2 were confirmed by MTA2 truncation and mutation. The effect of MTA1 overexpression on the intrinsic inhibitor of NE, elafin, was detected by qRT-PCR, immunoblotting and treatment with inhibitors.

**Results:**

MTA1 overexpression inhibited, while MTA2 promoted the metastasis of ZR-75-30 cells in vitro. MTA1 overexpression downregulated MTA2 expression at the protein level rather than the mRNA level. NE was predicted to cleave MTA2 and was responsible for MTA1 overexpression-induced MTA2 degradation. NE was found to cleave MTA2 in the C-terminus at the 486, 497, 542, 583 and 621 sites. MTA1 overexpression activated NE by downregulating elafin in a histone deacetylase- and DNA methyltransferase-dependent manner.

**Conclusions:**

MTA1 and MTA2 play opposing roles in the metastasis of ZR-75-30 luminal B breast cancer cells in vitro. MTA1 downregulates MTA2 at the protein level by epigenetically repressing the expression of elafin and releasing the inhibition of neutrophil elastase, which cleaves MTA2 in the C-terminus at multiple specific sites.

**Electronic supplementary material:**

The online version of this article (10.1186/s12964-019-0318-6) contains supplementary material, which is available to authorized users.

## Background

Breast cancer is the most prevalent cancer women suffer from worldwide and is estimated to be the cancer with the highest morbidity and second highest mortality among women in the United States [1]. Breast cancer is a highly heterogeneous cancer; therefore, it is classified into different subtypes, which include normal breast, basal-like, claudin-low, HER2 positive (HER2+), luminal A and luminal B cancers, according to the histological features and molecular traits of the cancer [2, 3]. Luminal B breast cancer shows more aggressive phenotypes than luminal A breast cancer and higher insensitivity to neoadjuvant chemotherapy than basal-like and HER2+ breast cancers, leading to its obstinacy and poor prognosis of patients [2, 4]. In addition, luminal B breast cancer tends to metastasize, increasing the mortality of luminal B breast cancer patients [2, 5]. In fact, breast cancer metastasis is the major lethal factor that affects patient mortality [6].

To overcome the problem of breast cancer metastasis, a 13762NF rat mammary adenocarcinoma metastasis model was established, and the first member of the metastasis associated protein (MTA) family, MTA1, was discovered by differential cDNA library screening in 1994 [7]. The other two members of the MTA family, MTA2 and MTA3, were identified afterwards [8, 9]. Subsequent studies have revealed that MTA1, MTA2 and MTA3 are constitutive components of nucleosome remodeling and histone deacetylase (NuRD) complex [10–12]. The NuRD complex possesses multiple activities, such as histone deacetylase activities and methyl-CpG-binding-related activities, because of the composition of histone deacetylase 1/2 (HDAC1/2) and methyl-CpG-binding domain protein 2/3 (MBD2/3), respectively [10, 13]. MTA1 and MTA2 have both been reported to be pivotal for epithelial-mesenchymal transition (EMT) and metastasis of breast cancer, while MTA3 has been reported to inhibit EMT [12, 14–16]. EMT is a critical step among a sequence of discrete steps that cancer cells undergo to achieve metastasis [17, 18]. During EMT, the EMT-inducing factors Snail, Slug, TWIST and ZEB1 are upregulated, the epithelial molecule E-cadherin is downregulated and the mesenchymal molecule N-cadherin is upregulated [17, 18].

Both being potent breast cancer metastasis-promoting factors of the MTA family, the difference and relationship between MTA1 and MTA2 remain an enigma because of insufficient investigations [19]. In this study, we aimed to examine the different roles that MTA1 and MTA2 may play in breast cancer metastasis and investigate their relationship. We investigated the roles of MTA1 and MTA2 in the metastasis of the ZR-75-30 luminal B breast cancer cell line [20, 21], and we found that the overexpression of MTA1 inhibits the metastasis of ZR-75-30 cells by downregulating MTA2, which has not been clarified previously [19]. Mechanistically, the overexpression of MTA1 transcriptionally downregulated the intrinsic inhibitor of the neutrophil elastase (NE), elafin, to promote the degradation of MTA2 that was mediated by NE, therefore inhibiting the metastasis of ZR-75-30 cells in vitro. Overall, our study provided direct evidence for the different roles that MTA1 and MTA2 play in breast cancer cell metastasis and clarified the mechanism by which MTA1 downregulates MTA2 at the protein level.

## Methods

### Cell culture and stable cell line establishment

293FT cells (Thermo Fisher Scientific) were cultured in Dulbecco’s Modified Eagles Medium supplemented with 10% fetal bovine serum (FBS) and 1% antibiotics (penicililin and streptomycin) HeLa cells (Type Culture Collection Cell Bank, Chinese Academy of Sciences) and ZR-75-30 cells (a kind gift from the Department of Breast Surgery, Fudan University Shanghai Cancer Center) were cultured in RPMI 1640 medium containing 10% FBS and 1% antibiotics. MDA-BM-231 cells (a kind gift from the Department of Breast Surgery, Fudan University Shanghai Cancer Center) were cultured in Leibovitz’s L-15 medium supplemented with 10% FBS and 1% antibiotics. All cells were maintained at 37 °C in a humidifed atmosphere containing 5% CO_2_ in a cell incubator (Thermo Fisher Scientific). ZR-75-30-Vector/MTA1/MTA2 stable cell lines were established by infection of lenti-viruses and selection by flow cytometry on MoFlo™ XDP (Beckman Coulter). ZR-75-30-shMTA2-NC/#1/#2, ZR-75-30-shElafin-NC/#1/#2 were established by infection of lenti-viruses and selection by puromycin. ZR-75-30-MTA1-shNE-NC/#1/#2 stable cell lines were established on the basis of ZR-75-30-MTA1 by infection of lenti-viruses and selection by puromycin.

### Antibodies and reagents

Anti-β-Actin antibody (sc-47,778), anti-MTA1 antibody (sc-17,773) and anti-MTA2 antibody (sc-55,566) were purchased from Santa Cruz Biotechnology (Dallas, TX, USA). Anti-Neutrophil Elastase antibody (ab131260) and anti-Elafin/Skalp antibody (ab46774) were purchased from Abcam (Cambridge, MA, USA). HA-Tag (C29F4) Rabbit mAb (#3724) was purchased from Cell signaling technology (Beverly, MA, USA). Monoclonal ANTI-FLAG® M2 antibody (F3165) was purchased from Merck Sigma company (Burlington, MA, USA). Anti-DDDDK-tag mAb (M185-3 L) was purchased from MBL International Corporation (Woburn, MA, USA). HRP conjugated secondary antibodies Goat anti-mouse IgG (H + L), HRP conjugate (SA00001–1) and Goat anti-rabbit IgG (H + L), HRP conjugate (SA00001–2) were purchased from Proteintech Group (Rosemont, IL, USA). Fluorescent secondary antibodies Goat anti-Rabbit IgG (H + L) Highly Cross-Adsorbed Secondary Antibody, Alexa Fluor 488 (A-11034) and Goat anti-Mouse IgG (H + L) Highly Cross-Adsorbed Secondary Antibody, Alexa Fluor 594 (A-11032) and lipofectamine 3000 (L3000015) were purchased from Thermo Fisher Scientific (Waltham, MA, USA). Puromycin (P8230) was purchased from Solarbio biotechnology (Shanghai, China). Cycloheximide (CHX, HY-12320) was purchased from MedChemExpress (Monmouth Junction, NJ, USA). Histone deacetylases (HDAC) inhibitor suberoylanilide hydroxamic acid (SAHA, also known as Vorinostat, MK0683; S1047), Sivelestat (S8136), MG132 (S2619) and Azacitidine (AZA, S1782) were purchased from Selleck Chemicals (Shanghai, China).

### Plasmids

To construct core plasmids overexpressing MTA1/MTA2, CDSs of MTA1/MTA2 were inserted into pCDH-EF1-MCS-T2A-copGFP. shRNA plasmids targeting MTA2/NE/Elafin were purchased from Shanghai Genechem. To construct FLAG-tagged MTA1/NE over-expressing plasmids, the CDS of MTA1/NE (without stop codon) were subcloned into pCMV-C-FLAG vector. To construct HA-tagged MTA2 and HA-tagged MTA2 truncated N-terminal and C-terminal mutants, specific primers were designed to subclone corresponding sequences from MTA2 (without stop codon) into pCMV-C-HA vector. For site mutation, specific mutation primers were designed and corresponding plasmids were mutated according to the manufacture of KOD-Plus-Mutagenesis Kit (SMK-101, TOYOBO, Japan). Mutations were confirmed by sequencing the plasmids. All the primers used to construct truncated mutants and point mutations are in Additional file 1: Table S1.

### Lentiviral particles production

To generate lentiviruses, core plasmids that overexpress or knockdown genes were co-transfected into 293FT cells with packaging plasmid psPAX2 and envelope plasmid pMD2.G at a ratio of 4:3:1 by Neofect™ DNA transfection reagent (TF201201, Neofect biotech, Beijing, China). After 48 h of incubation, lentiviral particles were harvested.

### Transfection

To detect the effect of MTA1 expression on the migration and invasion of MDA-MB-231 cells, MDA-MB-231 cells were transfected with pCDH-EF1-MCS-T2A-copGFP-MTA1 or empty vector as a control by lipofectamine 3000 as the manual instructed and cells were seeded for transwell assays after 36 h of transfection. After 48 h, cells were harvested for immunoblotting. To detect the effect of overexpression of MTA1 to MTA2 and its mutants, pCMV-C-FLAG-MTA1 and corresponding plasmids that overexpress MTA2 mutants were co-transfected into 293FT cells by Neofect™ DNA transfection reagent. For instance, 1250 ng pCMV-C-FLAG empty vector and 1250 ng pCMV-C-HA-MTA2 were co-transfected to 293FT cells seeded in a well of 6 well plate as the control group and 1250 ng pCMV-C-FLAG-MTA1 and 1250 ng pCMV-C-HA-MTA2 were co-transfected in the same as the experimental group. After 48 h of incubation, transfected cells were harvested for immunoblot. Simlar transfections were conducted for other cells.

### Immunoblot

To detect the levels of proteins in stable cell lines, the same amount of stable control cells and stable overexpression/knockdown cells were seeded in wells of 6 well plates. After 24 h incubation, cells were lysed by 1.25 × SDS loading buffer containing 1% β-mercaptoethanol. For transfected 293FT cells, after transfection, cells were incubated for 48 h, and then were lysed as above. Cell lysates were then heated in a heating block for 10 min at 100 °C with vigorous shaking. Same amount of heated cell lysates was loaded for SDS-PAGE respectively, followed by being transferred to PVDF membranes. Membranes were washed for 3 times with TBST and then blocked with 5% non-fat milk diluted with TBST at room temperature for 2 h. Primary antibodies diluted with 5% non-fat milk/TBST were incubated with blocked membranes for 2 h at room temperature or overnight at 4 °C on a gentle shaking platform. Membranes were washed with TBST for 3 times and then incubated with secondary antibodies conjugated with HRP diluted with 5% non-fat milk/TBST for 45 min at room temperature. Finally, membrane was washed by TBST for 3 times and developed with Immobilon Western Chemiluminescent HRP Substrate (WBKLS0500, MerckMillipore, (Burlington, MA, USA)). Images were captured with ImageQuant™ LAS 4000 biomolecular imager (GE Healthcare).

### Protein stability assay

To validate the effect of MTA1 to the stability of MTA2 protein, ZR-75-30-Vector and ZR-75-30-MTA1 cells were seeded and treated with Cycloheximide (CHX) at a final concentration of 20 μM for 0, 10, 25, 90, 240, 360 min respectively. Then cells were harvested followed by immunoblot detection.

### Quantitative RT-PCR (qRT-PCR)

Total RNA was extracted from cells as the manufacture of TRIzol reagent (10296010, Thermo Fisher Scientific (Waltham, MA, USA)) indicated. RNAs were reversely transcribed into cDNA using PrimeScript™ RT Master Mix (RR036A, Takara Bio Inc. (Japan)). qRT-PCR assay was conducted using SYBR® Premix Ex Taq™ II (Tli RNaseH Plus) (RR820Q, Takara Bio Inc.(Japan)) on a QuantStudio™ 7 Flex Real-Time PCR System platform (Thermo Fisher Scientific (Waltham, MA, USA)) following standard protocols. The expression of β-actin was set as endogenous control and the expressions of genes were calibrated to that of corresponding control cells. Data analyses were conducted by QuantStudio Real-Time PCR Software. RQ values (relative quantified value of mRNA expression) were calculated by the same software followed by two-tailed Student’s t test to determine significant differences between two groups. *P* < 0.05 was considered statistically significant. All the primers designed for qRT-PCR are in Additional file 1: Table S1.

### Immunocytochemistry

HeLa cells were seeded on the sterilized round coverslips that were inserted in to a well of a 24 well plate. After 12 h growth, cells were transfected with pCMV-C-HA-MTA2 + pCMV-C-FLAG plasmids or pCMV-C-HA-MTA2 + pCMV-C-FLAG-NE plasmids by Neofect reagent. Similar transfections were conducted for other cells. After 48 h of transfection, cells were fixed with 4% paraformaldehyde at room temperature for 30 min. Cells were then permeabilized on ice with 0.2% Triton X-100/PBS for 5 min, followed by washing 3 times using PBS. Then cells were blocked with 1%BSA/PBS at room temperature for 1.5 h and primary antibodies diluted with 1% BSA/PBS were applied for an incubation with cells at room temperature for 2 h. Cells were then washed by PBS for 3 times to wash off unbounded antibodies and incubated with fluorescent secondary antibodies at room temperature for 1.5 h. Then cells were washed and the coverslips were mounted with mounting medium containing DAPI. After an incubation at 4 °C for 15 min, slides were observed and images were captured at Leica TCS-SP5 confocal system.

### Cell migration and invasion assays

For cell migration assay, cells were harvested, washed by PBS for twice, resuspended by DMEM medium without FBS and counted by Vi-CELL XR (Beckman Coulter). Transwell (8 μm, 353097, Corning) chambers were inserted to corresponding wells in 24 well plate that already contain 500 μL DMEM with 10% FBS. For stable cell lines established on ZR-75-30 cells, without noticing, 2 × 10^4^ cells were added to the upper chamber and incubated for 24 h. For cell invasion assays, 60 μL matrigel (Matrigel™ Basement Membrane Matirx, 356,234, BD (Franklin Lakes, NJ, USA)) diluted with DMEM at a ratio of 1:10 was added to the upper chamber. The set was balanced at 4 °C for 30 min and then moved into cell incubator overnight to dry the gel. Similar with cell migration assay, cells were harvested, washed, resuspended and counted. 4 × 10^4^ cells were added to the matrigel precoated upper chamber under which the well contains 500 μL DMEM with 10% FBS, and incubated for 48 h. For migration and invasion assays performed on MDA-MB-231 cells or ZR-75-30-MTA1 cells, after 36 h of transfection, 4 × 10^4^ and 8 × 10^4^cells were added to the upper chamber and incubated for 24 h and 48 h respectively. For both cell migration assay and invasion assay, after incubation, medium of the upper chambers were discarded and cells migrated to the lower chamber were fixed with 4% paraformaldehyde at room temperature for 30 min, stained with 0.5% crystal violet at room temperature for 30 min, and washed by PBS for 3 times. Cells remained on the upper chamber were erased gently with a cotton-tipped swap. Cells were observed and images were captured at an Olympus microscope system. Migrated and invaded cells were then counted in randomly chosen three out of eight equally divided areas, followed by two-tailed Student’s t test to determine significant differences between two groups. *P* < 0.05 was considered statistically significant.

### Luminal B breast cancer tissue samples

Twenty paraffin-embedded tissue samples were derived from 20 luminal B breast cancer patients diagnosed and treated at the Department of Breast Surgery, The Third Hospital of Nanchang, Jiangxi Province, China. The Ethics Committee at The Third Hospital of Nanchang approved the utilization of samples, and all patients signed the informed consent form.

## Immunohistochemistry (IHC) staining assay

To detect the protein level of MTA1 and MTA2 in luminal B breast cancer patients, IHC was performed. Briefly, slices were deparaffinized in xylol. 10 mM sodium citrate (pH 6.0) was used for antigen retrieval, and endogenous peroxidase activity was eliminate with 3% H_2_O_2_. Then the slices were blocked with 1% bovine serum albumin/phosphate-buffered saline. Slices were incubated with anti-MTA1 antibody (sc-17,773, 1:100) or anti-MTA2 antibody (sc-55,566, 1:100) and placed in a humidified chamber and at 4 °C overnight. Reactions were developed using GTvision TM III (Gene Technology, Shanghai, China) and counterstained with 10% hematoxylin. At last, the stained slices were dehydrated and mounted with resinene. The staining indexes (0–12) for MTA1/MTA2 was obtained as the staining intensity (negative (0); weak (1); moderate (2); and strong (3)) multiplied by the proportion of positive staining ((0–25% (1); 25–50% (2); 50–75% (3); and 75–100% (4)).

## Results

### MTA1 overexpression inhibited the metastasis of ZR-75-30 luminal B breast cancer cells in vitro

**Fig. 1:**
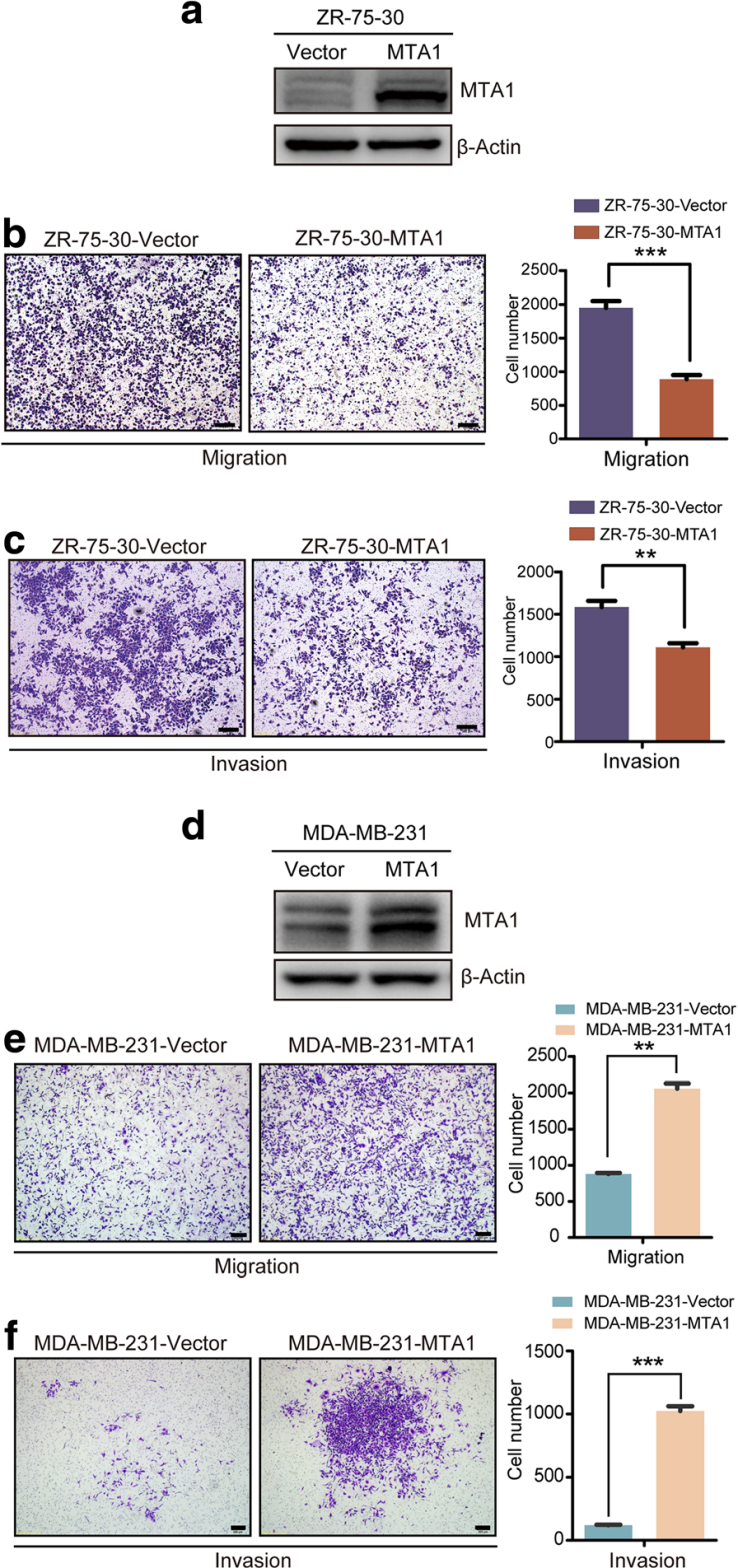
MTA1 overexpression inhibited the metastasis of ZR-75-30 cells. **a** MTA1 was overexpressed in ZR-75-30-MTA1 stable cell line compared to its control cell line ZR-75-30-Vector. Detected by immunoblot. **b** Overexpression of MTA1 significantly inhibited the migration and (**c**) invasion of ZR-75-30 cells in vitro. Detected by transwell migration and invasion assays. 30,000 cells migrated for 24 h. Scale bars = 200 μm, mean ± SD, *n* = 3,***P < 0.001, **P < 0.01, t test. **c** MTA1 was overexpressed in MDA-MB-231 cells compared with control cells. **d** Overexpression of MTA1 significantly promoted the migration and (**e**) invasion of MDA-MB-231 cells in vitro. Scale bars = 200 μm, mean ± SD, *n* = 3,***P < 0.001, **P < 0.01, t test

To investigate the role of MTA1 in luminal B breast cancer metastasis, we first established two stable cell lines, ZR-75-30-MTA1 overexpressing MTA1 and ZR-75-30-Vector as a control. MTA1 overexpression was confirmed by immunoblotting (Fig. 1a). Then, we evaluated the metastatic potential of these cell lines by Transwell chamber cell migration and invasion assays. Surprisingly, contradictory to the well-established roles of MTA1, the overexpression of MTA1 significantly inhibited the migration and invasion of ZR-75-30 cells in vitro (Fig. 1b and c).

We hypothesized that the metastasis-inhibitory role of MTA1 in ZR-75-30 cells may due to the particularity of ZR-75-30 cell line. To testify our hypothesis, we overexpressed MTA1 in MDA-MB-231 cells, a triple negative breast cancer cell line (Fig. 1d). The results of migration and invasion assays indicated that overexpression of MTA1 significantly promoted the migration and invasion of MDA-MB-231 cells in vitro (Fig. 1e and f). The results showed that MTA1 played different roles in the migration and invasion of different breast cancer cells in vitro.

### Overexpression of MTA1 downregulated MTA2, which promoted the metastasis of ZR-75-30 cells

**Fig. 2:**
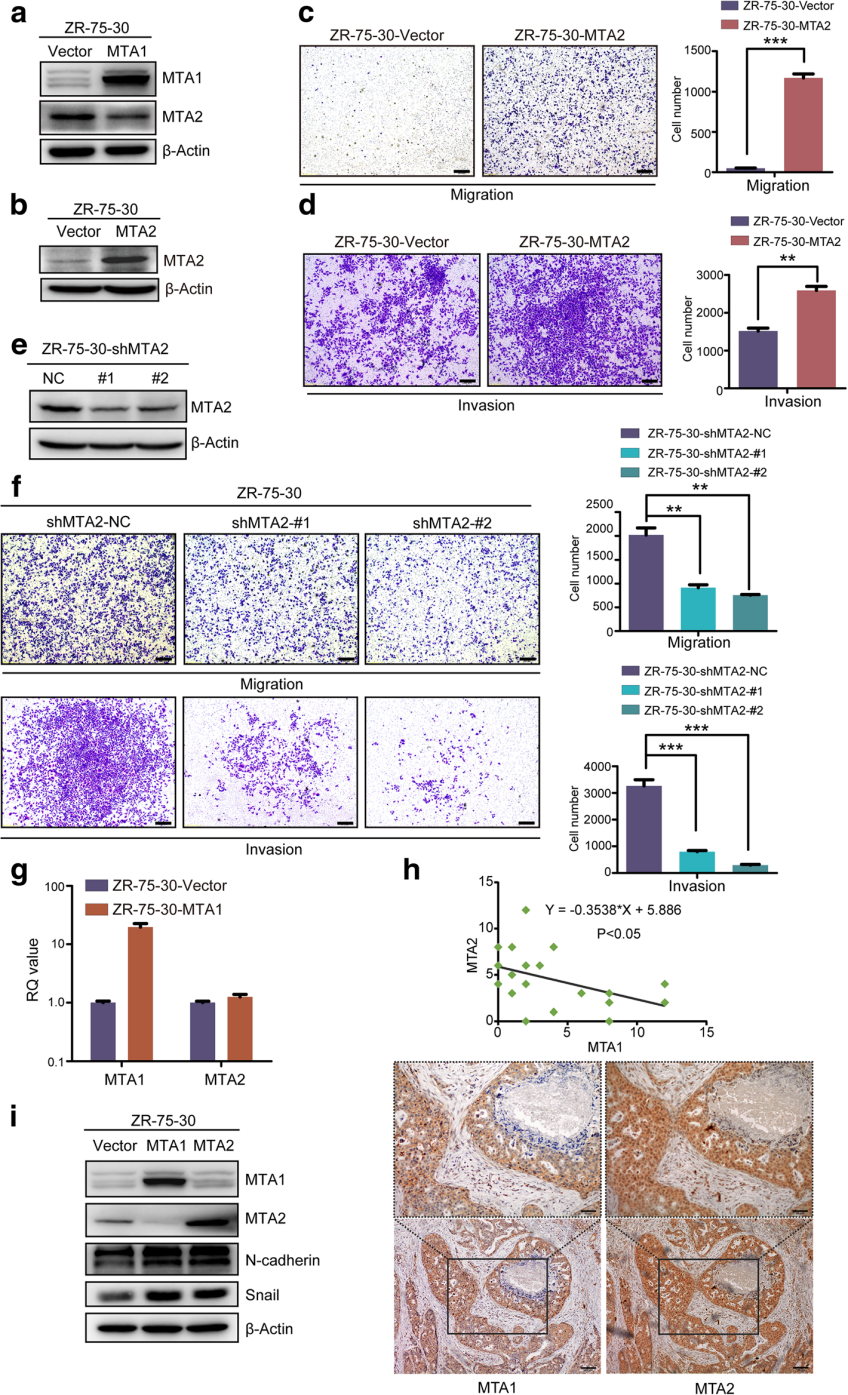
Overexpression of MTA1 downregulated MTA2 which promotes the metastasis of ZR-75-30 cells. **a** MTA2 was downregulated by MTA1 overexpression. **b** MTA2 was overexpressed in ZR-75-30-MTA2 cell line. **c** Overexpression of MTA2 significantly promoted the migration and (**d**) invasion of ZR-75-30 cells in vitro. 50,000 cells migrated for 20 h. 40,000 cells, invaded for 2.5d. Scale bars = 200 μm, mean ± SD, *n* = 3,**P < 0.01, ***P < 0.001, t test. **e** MTA2 was knocked down by 2 shRNAs specifically targeting MTA2. **f** MTA2 knockdown significantly inhibited the migration and invasion of ZR-75-30 cells. 35,000 cells and 50,000 cells migrated for 36 h and invaded for 3.5d respectively. Scale bars = 200 μm, mean ± SD, *n* = 3,**P < 0.01,***P < 0.001, t test. **g** Overexpression of MTA1 did not repress the transcription of MTA2. Detected by qRT-PCR. Mean ± SD, *n* = 3, RQ value: relative quantified value of mRNA expression. **h** The protein level of MTA1 and MTA2 are negatively correlated in tissues derived from luminal B breast cancer patients. Detected by IHC. Scale bars in upper box = 50 μm, scale bars in lower box = 100 μm. **i** Both overexpression of MTA1 and MTA2 increased EMT landmarks Snail and N-cadherin

The overexpression of MTA1 has been shown to downregulate the expression of MTA2 at the protein level instead of by transcriptional repression in breast cancer cells [19]. Because of the role of MTA2 in breast cancer metastasis, we hypothesized it's the same reason for MTA1-induced metastasis inhibition. To verify our hypothesis, we evaluated the protein expression level of MTA2 in ZR-75-30-MTA1 cells by immunoblotting and found that it decreased (Fig. 2a).

To validate the role of MTA2 in ZR-75-30 metastasis, we established a ZR-75-30-MTA2 cell line using the same system used to establish the ZR-75-30-MTA1 cell line (Fig. 2b). MTA2 overexpression significantly enhanced ZR-75-30 cell metastasis in vitro (Fig. 2c and d). Knocking down MTA2 using two shRNAs targeting MTA2 significantly inhibited the metastasis of ZR-75-30 cells in vitro (Fig. 2e and f).

To further verify our hypothesis, we measured the mRNA expression level of MTA2 in ZR-75-30-Vector and ZR-75-30-MTA1 cells by qRT-PCR. In accordance with previous studies, the overexpression of MTA1 did not repress MTA2 transcription (Fig. 2g).To further determine if there’s negative correlation between the protein levels of MTA1 and MTA2 in luminal B breast cancer patients, we performed Immunohistochemistry (IHC) staining for MTA1 and MTA2 on slices prepared from serial section. Scoring of the IHC staining of MTA1 and MTA2 indicated a negative correlation between the protein level of MTA1 and MTA2 in luminal B breast cancer tissues (*P* < 0.05), and representative images are shown (Fig. 2h).

Both MTA1 and MTA2 have been reported to promote EMT and breast cancer metastasis. To investigate whether the different roles of MTA1 and MTA2 in ZR-75-30 cell metastasis are caused by the different roles of these factors in the EMT of ZR-75-30 cells, we evaluated the expression levels of the indicators of EMT (Snail and N-cadherin) and found that both MTA1 and MTA2 upregulated the expression of these indicators (Fig. 2i).

These data indicated that although both MTA1 and MTA2 promoted the EMT process in ZR-75-30 cells, the overexpression of MTA1 inhibited the metastasis of ZR-75-30 cells by decreasing MTA2 protein stability.

### Neutrophil elastase is responsible for MTA1-induced MTA2 degradation

**Fig. 3:**
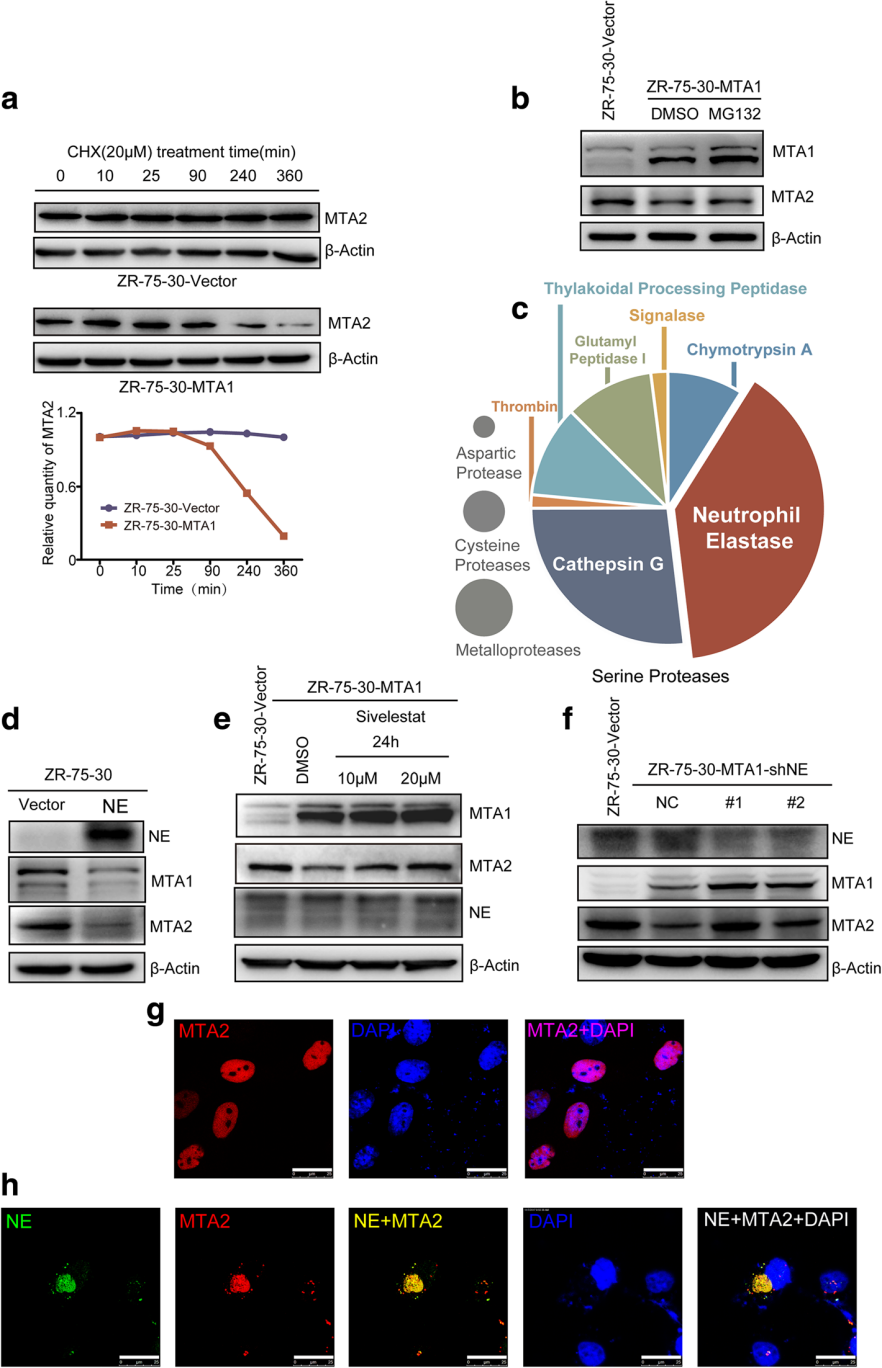
NE is responsible for MTA1 induced MTA2 downregulation. **a** Overexpression of MTA1 decreased the protein stability of MTA2. **b** Treatment of ZR-75-30-MTA1 cells with MG132 (5 μM, 12 h) increased the protein level of MTA1 but did not increase that of MTA2. **c** Schematic diagram of the proteases predicted to cleave MTA2 at PROSPER website.NE is predicted to have the most cleavage sites in MTA2. **d** Overexpression of NE downregulated MTA2 and MTA1. **e** NE specific inhibitor sivelestat treatment restored MTA2, and upregulated MTA1, in ZR-75-30-MTA1 cells in a dose dependent manner. **f** NE knockdown increased the protein level of MTA2 and MTA1. **g** MTA2 located exclusively in nuclei. Detected by ICC. Scale bars = 25 μm. **h** MTA2 and NE co-localized in cytoplasm. Scale bars = 25 μm

To further confirm the influence of MTA1 on the stability of MTA2, we conducted a protein stability assay using cycloheximide (CHX). The results showed that the overexpression of MTA1 significantly decreased the stability of MTA2 (Fig. 3a).To elucidate the mechanism by which MTA1 downregulates MTA2, we first treated ZR-75-30-MTA1 cells with MG132 (5 μM for 12 h) and detected an upregulation of MTA1 (Fig. 3b), which was reported to be regulated by the ubiquitin-proteasome pathway [22]. However, we did not detected the upregulation of MTA2 by immunoblotting (Fig. 3b). Further, we predicted enzymes that may cleave MTA2 by its amino acid sequence using the PROtease Specificity Prediction servER [23] (PROSPER). MTA2 was predicted to be cleaved by four types of proteases, including aspartic proteases, cysteine proteases, metalloproteases and serine proteases, among which the serine protease neutrophil elastase (NE) was predicted to cleave MTA2 with the highest probability (Fig. 3c). To evaluate whether NE can degrade MTA2, we overexpressed NE in ZR-75-30 cells, and as shown in Fig. 3d, the overexpression of NE led to a decrease in MTA2, and suprisingly, a decrease in MTA1.

To verify whether NE is responsible for MTA1-induced MTA2 degradation, we first treated ZR-75-30-MTA1 cells with a specific NE inhibitor, sivelestat, with a concentration gradient or with the solvent of sivelestat (DMSO) as a control for 24 h. As shown in Fig. 3e, the treatment of ZR-75-30-MTA1 cells with sivelestat restored MTA2 in a dose-dependent manner. Furthermore, we observed that the protein expression level of MTA1 also increased in the same manner as MTA2. The increase in MTA1 was not expected, but it further indicated that NE is responsible for MTA1-induced deregulation of MTA2 because the increase in MTA1 did not further downregulate MTA2. To further confirm the role of NE in MTA1-induced MTA2 degradation, we knocked down NE in ZR-75-30-MTA1 cells. The immunoblot results showed that knocking down NE led to a similar result as the sivelestat treatment (Fig. 3f).

As a serine protease that is stored in large quantities in neutrophil cytoplasmic azurophilic granules [24, 25], the interaction of NE with MTA2 that is located in the nucleus needed to be elucidated. Therefore, we cotransfected HA-tagged MTA2 with an empty vector or with FLAG-tagged NE into HeLa cells and analyzed the cellular location of these molecules by immunocytochemistry (ICC). HA-tagged MTA2 was exclusively detected in nuclei, while it was colocalized with NE in the cytoplasm (Fig. 3g and h), providing evidence of an interaction between MTA2 and NE.

### MTA1 repressed the transcription of elafin, an intrinsic NE inhibitor, in an HDAC- and/or DNMT-dependent manner

**Fig. 4:**
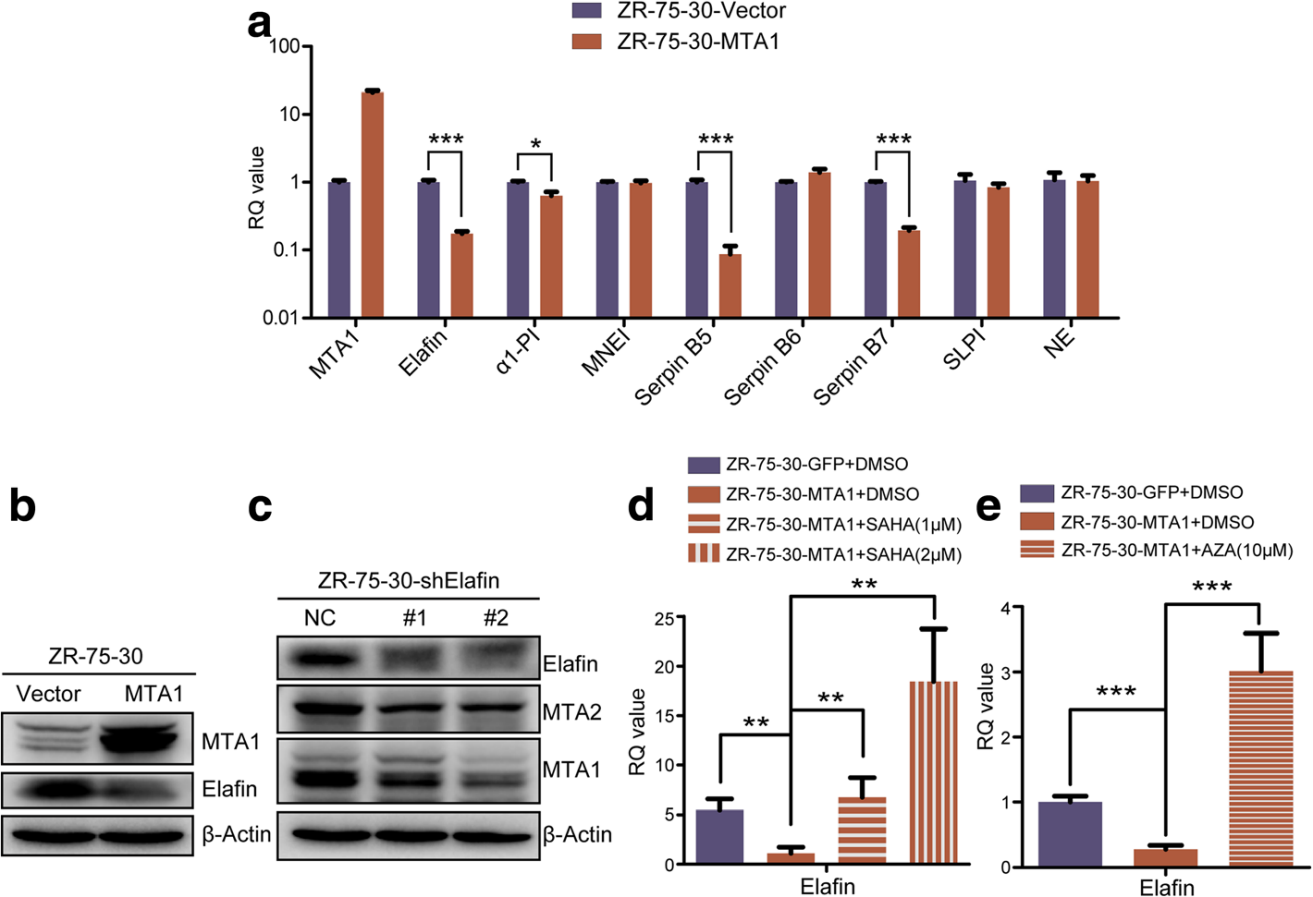
MTA1 overexpression significantly inhibited the transcription of the intrinsic inhibitor of NE, elafin, in a HDAC and/or DNMT dependent manner. **a** Overexpression of MTA1 significantly downregulated the mRNA level of intrinsic inhibitors of NE, elafin and α1-PI, and serine protease inhibitors Serpin B5 and Serpin B7, but didn’t significantly change the transcription of NE, MNEI, Serpin B6, and SLPI. Mean ± SD, *n* = 3, *P < 0.05, ***P < 0.001, t test. **b** MTA1 overexpression decreased the protein level of elafin. **c** Knocking down of elafin decreased MTA2 and MTA1 at protein level. **d** HDAC inhibitor and **e** DNMT inhibitor treatments increased the mRNA level of elafin. ZR-75-30-MTA1 cells were treated with HDAC inhibitor SAHA and DNMT inhibitor, AZA, for 48 h, after which the mRNA level of elafin significantly elevated, even to a value higher than that of ZR-75-30-Vector. Mean ± SD, *n* = 3, **P < 0.01, ***P < 0.001, t test

To further exploit the association between MTA1 and serine protease NE, we tested whether MTA1 could transcriptionally regulate NE, its intrinsic inhibitors and some other intrinsic serine protease inhibitors. The qRT-PCR results showed that MTA1 overexpression significantly repressed the transcription of 2 intrinsic inhibitors of NE, elafin and α1-PI. Besides, the transcription level of serine protease inhibitors Serpin B5 and Serpin B7 were also significantly decreased following MTA1 overexpression (Fig. 4a). We chose the dramatically decreased NE inhibitor, elafin, for further investigation. Immunoblotting also showed a decrease in elafin at the protein level in ZR-75-30-MTA1 cells (Fig. 4b). To further confirm that the downregulation of elafin by MTA1 leads to MTA2 degradation, we knocked down elafin in ZR-75-30 cells with two shRNAs and found a decrease in MTA2 and MTA1 expression levels, in accordance with the results shown in Fig. 3.

As a constitutive component of the NuRD complex, MTA1 could epigenetically modulate gene expression. To explore the mechanism by which MTA1 regulates elafin, we treated ZR-75-30-MTA1 cells with a concentration gradient of an HDAC inhibitor, suberoylanilide hydroxamic acid (SAHA, which is also known as vorinostat) and the solvent (DMSO) as a control for 48 h. The qRT-PCR results showed that treatment with SAHA significantly upregulated elafin transcription in a dose-dependent manner, and the transcription level of elafin in ZR-75-30-MTA1 cells was even released to a level exceeded that of ZR-75-30-Vector cells after SAHA treatment, indicating a strong repression of elafin transcription by MTA1/HDACs (Fig. 4d). MTA1 and MBD2 (or MBD3) jointly participate in NuRD formation, and it has been reported that MTA1 forms a complex with DNMT to regulate gene expression. Therefore, we also hypothesized that MTA1 may regulate elafin transcription in a DNA methyltransferase (DNMT)-dependent manner. Treatment with a DNMT inhibitor, azacitidine (AZA), also led to a significant release of elafin transcription, indicating that MTA1 overexpression inhibited elafin transcription in a DNMT-dependent manner.

These results demonstrated that MTA1 overexpression led to the downregulation of elafin, an intrinsic inhibitor of NE, in an HDAC- and/or DNMT-dependent manner, thus impairing NE inhibition and promoting MTA2 degradation.

### NE cleaves MTA2 at its C-terminus

**Fig. 5:**
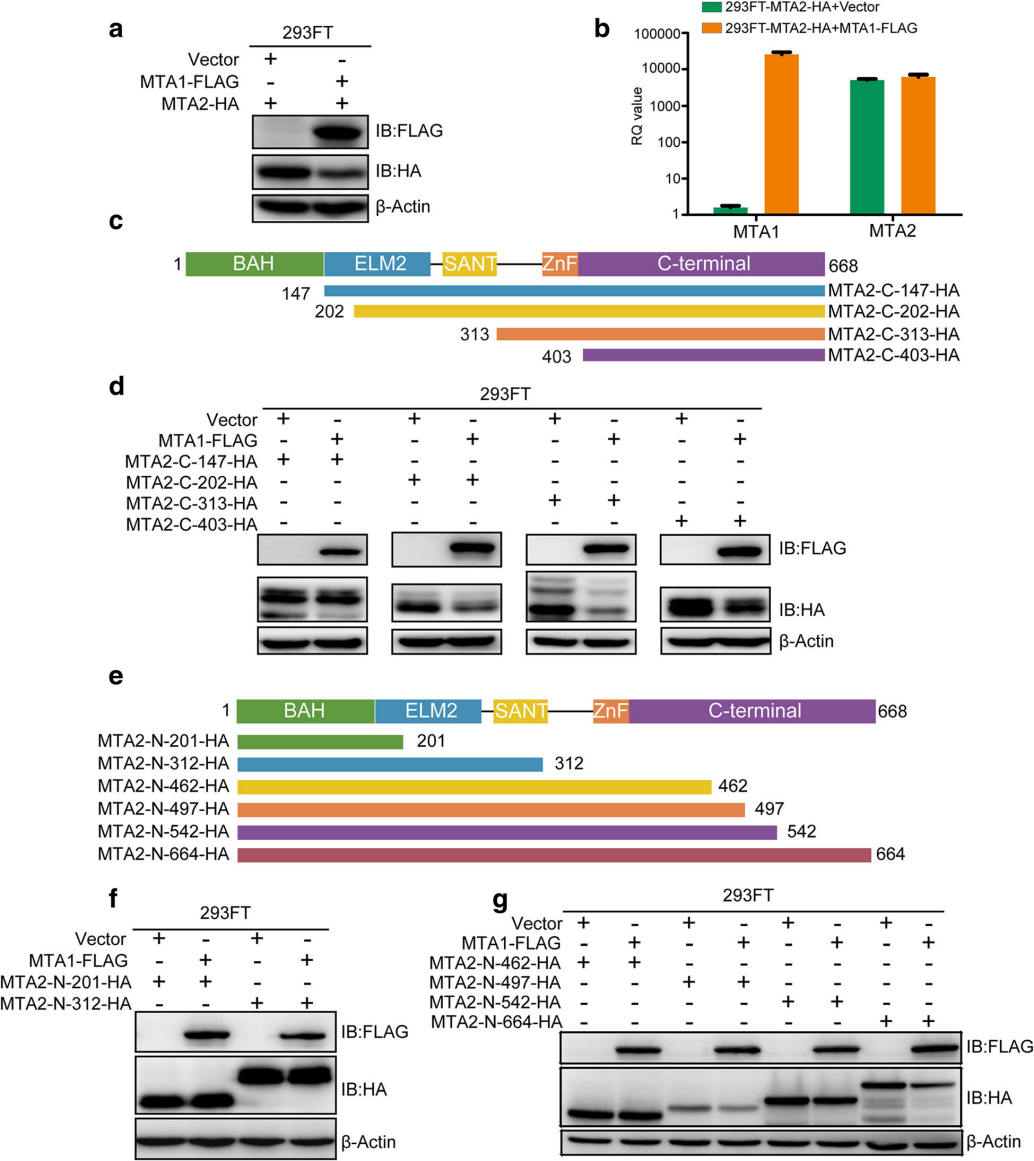
NE cleavage site(s) locate(s) in MTA2 at its C terminus. **a** FLAG-tagged MTA1 downregulated HA-tagged MTA2 in 293FT cells. **b** The expression of MTA1-FLAG did not decrease the mRNA level of MTA2-HA in 293FT cells. Mean ± SD, *n* = 3, t test. **c** Schematic diagram of the constructed C-terminal truncated mutants of MTA2 fused with HA tag. The full length of MTA2 contains 668aa. The C-terminal truncated mutant from 147aa to 668aa fused with HA tag was designated as MTA2-C-147-HA, and the rest were designated in a similar fashion. **d** MTA1-FLAG downregulated all 4 C-terminal truncated mutants of MTA2. **e** Schematic diagram of the constructed MTA2 N-terminal truncated mutants. **f** MTA2-N-201-HA, MTA2-N-312-HA and (**g**) MTA2-N-462-HA instead of MTA2-N-497-HA and longer N-terminal truncated mutants of MTA2 could not be downregulated by MTA1-FLAG

To identify the NE cleavage site(s) in MTA2, we established a detection system in 293FT cells. We transiently transfected plasmid combinations pCMV-C-HA-MTA2 + Vector and pCMV-C-HA-MTA2 + pCMV-C-FLAG-MTA1 into 293FT cells and detected the protein and mRNA expression levels of MTA1-FLAG and MTA2-HA using antibodies against the corresponding tags and specific primers, respectively. In accordance with the observations in ZR-75-30 cells, the overexpression of MTA1-FLAG also decreased MTA2-HA expression at the protein level rather than at the mRNA level in 293FT cells (Fig. 5a and b).

Because NE can modulate the function of proteins by specific cleavage, we hypothesized that NE may cleave MTA2 at the functional domains, including the bromo-adjacent homology (BAH) domain (1–144 aa), Egl-27 and MTA1 homology 2 (ELM2) domain (145–256 aa), SANT domain (263–315 aa), zinc finger (ZnF) domain (267–394 aa) and the C-terminus (395–668 aa) where its nuclear localization signals locate [26]. We constructed different C-terminal-truncated mutants of MTA2 fused with an HA tag that were designed based on the domains of MTA2, which were designated as MTA2-C-147-HA, MTA2-C-202-HA, MTA2-C-313-HA and MTA2-C-403-HA (Fig. 5c), and cotransfected them with FLAG-tagged MTA1 or an empty vector as the control into 293FT cells. The immunoblot results showed that all 4 C-terminal-truncated mutants of MTA2 were downregulated by the co-expression of MTA1-FLAG (Fig. 5d).

The result shown in Fig. 5d indicated that MTA2 may be cleaved in a random pattern or may be cleaved at the C-terminus by NE. To validate this hypothesis, we constructed vectors containing different N-terminal truncated mutants of MTA2 fused with an HA tag that were designed based on the domains of MTA2 (designated MTA2-N-201-HA and MTA2-N-312-HA) or the predicted cleavage sites of NE (designated MTA2-N-462-HA, MTA2-N-497-HA, MTA2-N-542-HA and MTA2-N-664-HA) (Fig. 5e). We found that both MTA2-N-201-HA and MTA2-N-312-HA could not be downregulated by the co-expression of MTA1-FLAG (Fig. 5f). In addition, in other N-terminal truncated mutants, MTA2-N-462-HA could not be downregulated by the co-expression of MTA1; however, the MTA2-N-497-HA, MTA2-N-542-HA and MTA2-N-664-HA mutants were downregulated when MTA1-FLAG was co-expressed (Fig. 5g). These results indicate that NE cleaves MTA2 at its C-terminus.

### Identification of NE cleavage sites in the MTA2 C-terminus

**Fig. 6:**
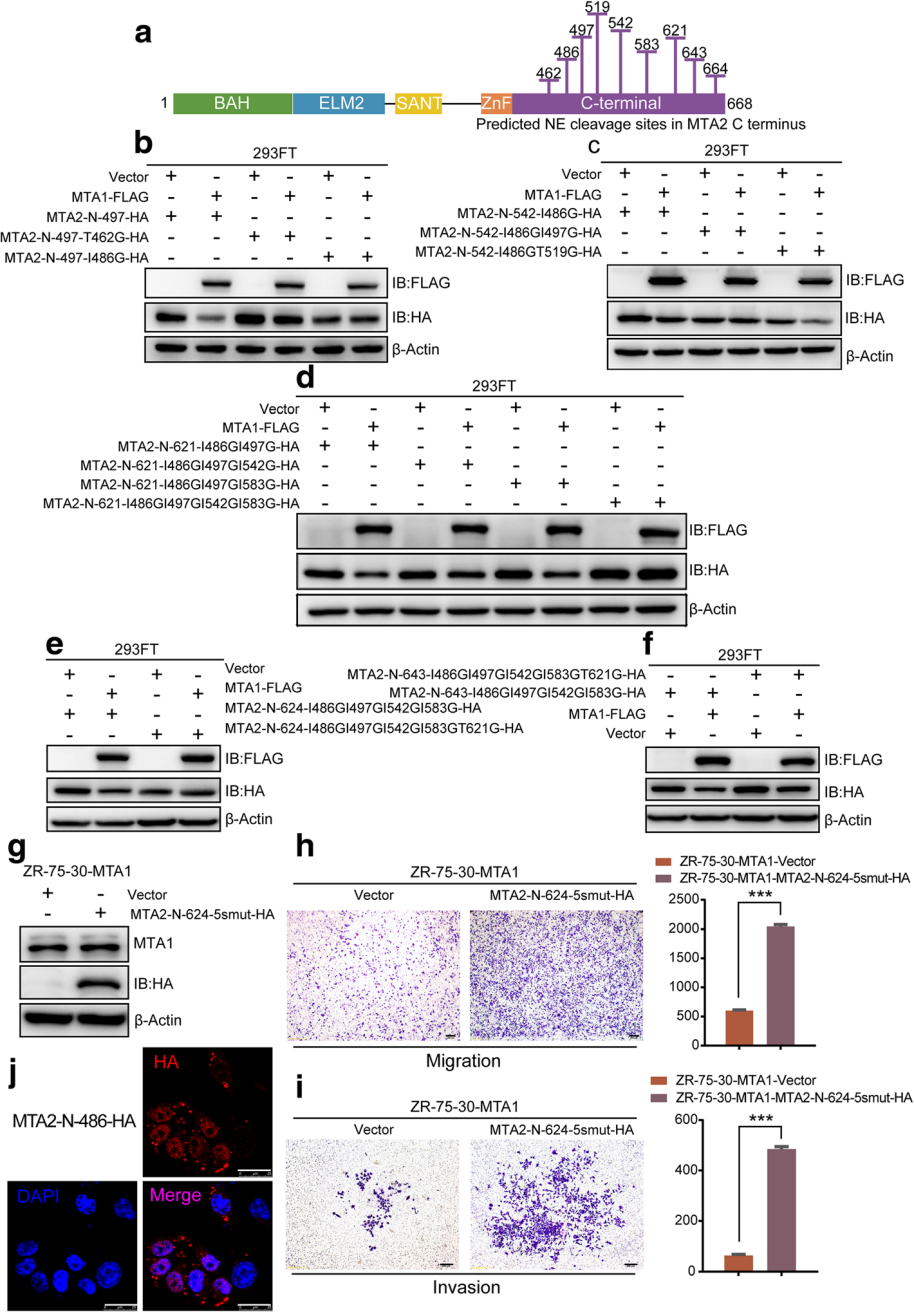
486, 497, 542, 583 and 621 sites were identified to be NE cleavage sites of MTA2. **a** Schematic diagram of the predicted 9 NE cleavage sites after 461 locating in MTA2 C-terminus. **b** Identification of 486, (**c**) 497 (**d**) 542, 583 and (**e**) 621 as NE cleavage sites. **f** Unpredicted NE cleavage site(s) may locate between 621 and 643 sites. **g** MTA2-N-624-I486GI497GI542GI583GT621G-HA, designated as MTA2-N-624-5smut-HA was overexpressed in ZR-75-30 cells. **h** Overexpression of MTA2-N-624-5smut-HA significantly promoted the migration and (**i**) invasion of ZR-75-30-MTA1 cells. Scale bars = 200 μm, mean ± SD, *n* = 3,***P < 0.001, t test. **j** MTA2-N-486-HA showed a tendency of diffusing into cytoplasm. Scale bars = 25 μm

As predicted by the PROSPER website, there are 9 potential NE cleavage sites located in the MTA2 C-terminus, including the 462, 486, 497, 519, 542, 583, 621, 643 and 664 sites (Fig. 6a). To identify NE cleavage sites in the MTA2 C-terminus, we constructed different N-terminal-truncated mutants either with or without point mutations to identify NE cleavage sites in the MTA2 C-terminus.

To validate whether 462 and/or 486 are/is NE cleavage site(s), we constructed an N-terminal truncated mutant of MTA2, MTA2-N-497-HA, and mutated 462 T to 462G (MTA2-N-497-T462G-HA) and 486I to 486G (MTA2-N-497-I486G-HA), respectively. The immunoblot results showed that when co-expressed with MTA1-FLAG, MTA2-N-497-HA was downregulated. The I486G mutation, but not the T462G mutation, impeded the downregulation process (Fig. 6b). This result indicates that the 486 site is one of the NE cleavage sites in the MTA2 C-terminus, while the 462 site is not. Similar strategies were applied to identify that 497 (Fig. 6c), 542, 583 (Fig. 6d) and 621 (Fig. 6e) are NE cleavage sites.

However, the MTA2-N-643-I486GI497GI542GI583GT621G-HA mutant was still downregulated by MTA1-FLAG co-expression (Fig. 6f), suggesting that there might be unpredicted NE cleavage site(s) between site 621 and site 643 or that there might be another delicate and complex underlying mechanism; therefore, additional cleavage sites were not verified.

To elucidate whether restoring MTA2 would increase the migration and invasion capacity of ZR-75-30-MTA1 cells, we transfected plasmids containing MTA2-N-624-I486GI497GI542GI583GT621G-HA (designated as MTA2-N-624-5smut-HA thereafter) or empty vector as a control into ZR-75-30-MTA1 cells (Fig. 6g). In vitro migration and invasion assays indicated that overexpression of MTA2-N-624-5smut-HA significantly promoted the migration and invasion of ZR-75-30-MTA1 cells (Fig. 6h and i).

To elucidate the co-localization of MTA2 and NE, we transfected plasmids expressing MTA2-N-486-HA into ZR-75-30 cells. The result of ICC detection indicated that the truncated MTA2 no longer showed a strong nucleus-located pattern as detected in Fig. 3g. It showed a tendency to diffuse into the cytoplasm (Fig. 6j). The result implies that NE cleavage at the C-terminus destroyed the nuclear localization signals of MTA2 and led to the cytoplasm location of MTA2.

## Discussion

As the first member of the MTA family, MTA1 has been linked to breast cancer metastasis since its discovery, and its crucial role in carcinogenesis and cancer metastasis has been revealed by important studies [7, 14, 15, 27–29]. Surprisingly, in our study, we found that MTA1 overexpression significantly inhibited the metastasis of ZR-75-30 luminal B breast cancer cells.

In accordance with previous reports, we found that overexpression of MTA1 promoted the migration and invasion of MDA-MB-231 cells in vitro. Besides, we found that both overexpression of MTA1 and MTA2 promoted EMT events in ZR-75-30 cells. However, both the migration and invasion of ZR-75-30 cells were impeded and promoted by MTA1 and MTA2 overexpression, respectively, showing the different roles MTA1 and MTA2 play in the metastasis of ZR-75-30 cells. The results are seemingly contradictory to the canonical roles of MTA1, but they are reasonable. It has been reported that MTA1 exhibited different expression and distribution patterns during the development of breast cancer in a mouse model, implying that the roles of MTA1 are altered sequentially [30]. Another member of MTA family, MTA3, which has been reported to inhibit the EMT of breast cancer by transcriptionally downregulating Snail [12], was reported to promote the progression of non-small cell lung cancer and hepatocellular carcinoma [31, 32], implying that the roles of MTA family members may change with different cancer types. Therefore, the inhibitory role of MTA1 in ZR-75-30 cells may be due to the specific context in this cell line or in luminal B breast cancer.

We also reported that MTA1 overexpression post-translationally, but not transcriptionally, downregulated MTA2 in ZR-75-30 cells. The same result has been reported in MCF-7 and T47D cells, but the mechanism has not been elucidated [19]. MTA2 shares 58% of its homology with MTA1 and exhibits functions that are similar to those of MTA1 [8]. In our study, both MTA1 and MTA2 evaluated the expression of Snail and N-cadherin. Recently, it has been shown that EMT, as a stage of metastasis, can be divided into different transition states and endow different metastatic capacities to cancer cells, suggesting that enhanced EMT does not necessarily indicate enhanced metastasis [33]. Furthermore, it has also been reported that MTA2 can alter the activity of Rho GTPase by regulating Rho GDIα in MDA-MB-231 breast cancer cells [34]. This observation indicated that the different results of ZR-75-30 cell metastasis that we obtained in our study may be due to the different attributes of MTA1 and MTA2 in cytoskeleton modulation. Elucidating the difference between MTA1 and MTA2 may lead to an improved understanding of breast cancer metastasis.

NE is a serine protease that is mainly present in specialized lysosomes (azurophil granules) of neutrophils [24, 25]. In breast cancer, it has been reported that NE can be secreted by breast cancer cells instead of normal breast cells, and a greater level of NE is associated with a more advanced stage and poor prognosis of breast cancer [35]. NE has also been reported to promote the metastasis of MDA-MB-231 breast cancer cells that can be inhibited by elafin [36]. In our study, we found that when co-expressed, NE and MTA2 were localized to the cytoplasm, suggesting that NE may cleave MTA2 in the cytoplasm. However, how co-expression of NE relocates MTA2 and exactly where they interact remains unknown. In addition, whether the interaction of NE with MTA2 is associated with its malignant roles in breast cancer and in what circumstances NE promotes or inhibits breast cancer metastasis remain interesting questions that require further exploration. In addition, beyond the downregulation of MTA2, we did not observe bands of the cleaved products of MTA2 by NE, which may suggest a more delicate and complex regulatory mechanism of MTA2 by NE.

To determine the link between NE and MTA2, we found that overexpression of MTA1 significantly inhibited the transcription of intrinsic inhibitors of NE, including α1-PI and elafin, and other serine protease inhibitors. MTA1 downregulated elafin in an HDAC- and DNMT-dependent/ NuRD complex-dependent manner. Elafin has been reported to inhibit the proliferation of breast cancer cells instead of normal cells [36], and during the tumorigenesis of breast cancer, elafin was shown to be downregulated in invasive breast cancer compared to normal mammary epithelium [37]. This result is consistent with the canonical role of MTA1 and is consistent with the results of our study. It can be speculated that at the initial stage of tumorigenesis, upregulated MTA1 downregulates elafin to promote breast cancer initiation, proliferation and progression. However, the roles of MTA1 and elafin seemingly change sequentially. Similar to the seemingly contradictory roles of MTA1, the residual expression of elafin in invasive breast cancer predicts a significantly poor prognosis of breast cancer patients: a tumor suppressor gene turned into an oncogene [37]. Our experimental results consistently indicated that the roles of oncogenes and anti-oncogenes, such as MTA1 and elafin, could be altered and that they could play different roles in different contexts.

## Conclusions

**Fig. 7:**
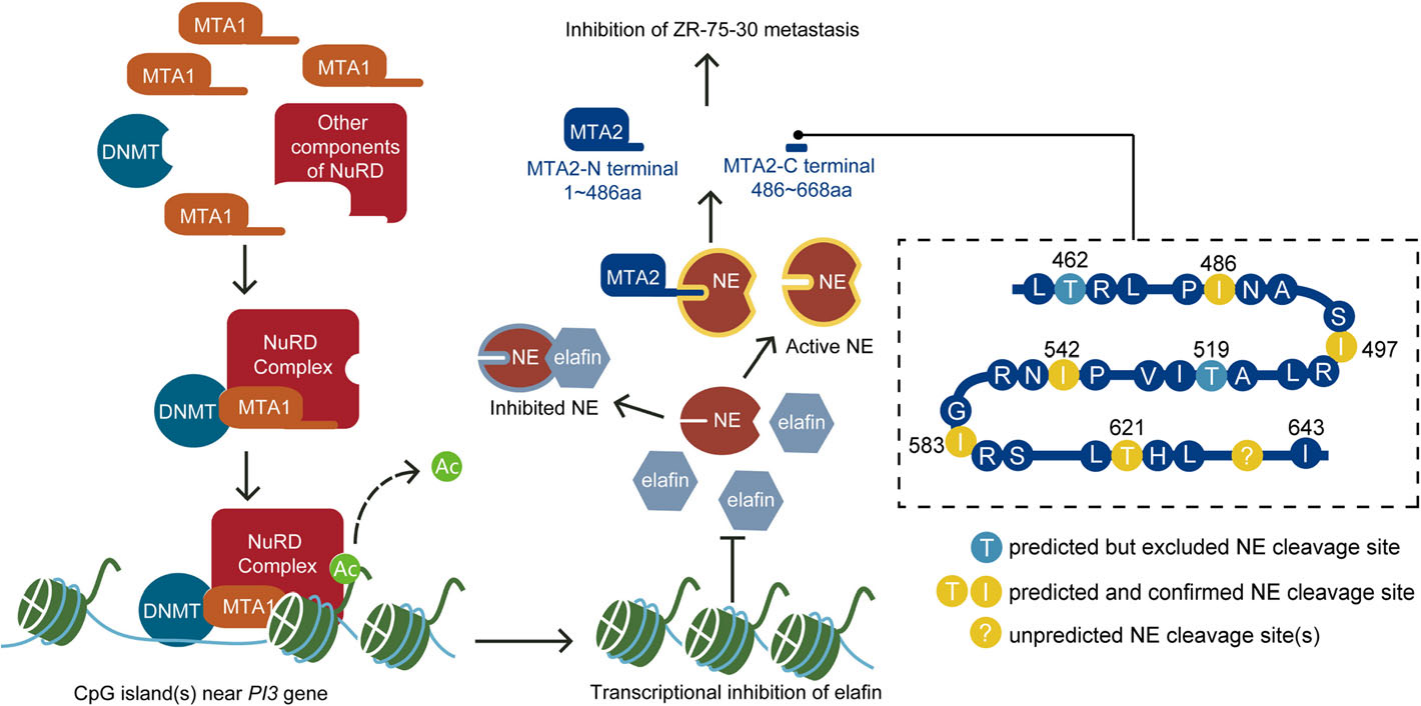
Schematic diagram of the mechanism by which MTA1 downregulate MTA2

Our study revealed the different roles that MTA1 and MTA2 play in ZR-75-30 metastasis and the mechanism underlying the downregulation of MTA2 by MTA1. MTA1 epigenetically inhibited the transcription of elafin, which decreased the inhibition of NE, and NE cleaved MTA2 in its C-terminus at the 486, 497, 542, 583, 621 sites to downregulate MTA2 posttranslationally, leading to the inhibition of the metastasis of ZR-75-30 cells in vitro (Fig. 7). These results provide an improved understanding of the difference and relationship between MTA1 and MTA2 and breast cancer metastasis as well as the flexible roles that genes may play in different contexts.

## Additional file

Additional file 1: **Table S1.** Main primers used in the study. (DOC 64 kb)

## Abbreviations

AZA: Azacitidine
BAH: Bromo-adjacent homology
CDS: Coding DNA sequence
CHX: Cycloheximide
DNMT: DNA methyltransferase
ELM2: Egl-27 and MTA1 homology 2
EMT: Epithelial-mesenchymal transition
FBS: Fetal bovine serum
HDAC: Histone deacetylase
HER2: Human epidermal growth factor receptor 2
ICC: Immunocytochemistry
MBD: Methyl-CpG-binding domain protein
MTA: Metastasis associated protein
NC: Negative control
NE: Neutrophil elastase
NuRD: Nucleosome remodeling and histone deacetylase
PROSPER: Protease specificity prediction server
qRT-PCR: Real-time quantitative reverse transcription polymerase chain reaction
RQ value: Relative quantified value of mRNA expression
SAHA: Suberoylanilide hydroxamic acid
SANT: Swi3, Ada2, N-Cor, and TFIIIB
SDS-PAGE: Sodium dodecyl sulfate–polyacrylamide gel electrophoresis
TBST: Tris-buffered saline and Tween 20
ZnF: znic finger
