# Supplementary material for: Overexpression of MTA1 inhibits the metastatic ability of ZR-75-30 cells in vitro by promoting MTA2 degradation
Source: Cell Commun Signal. 2019 Jan 14;17:4. doi: 10.1186/s12964-019-0318-6 (PMC6332694; doi:10.1186/s12964-019-0318-6)
Supplement: Supplementary file 1 — Table S1. Main primers used in the study. (DOC 64 kb) [file 12964_2019_318_MOESM1_ESM.doc]

Additional file 1 Table S1. Main primers used in the study

| Primers used for the establishment of plasmids containing truncated mutants and point mutations of MTA2 | Sequence (without restriction sites) |
| --- | --- |
| MTA2-C-147-F-primer | ATGattagagttggttgcaa |
| MTA2-C-202-F-primer | ATGacctttgcaagagccct |
| MTA2-C-313-F-primer | ATGacagaccggtatattca |
| MTA2-C-403-F-primer | ATGggactgaagaccccaac |
| MTA2-R-Primer | gtcctccaggacaata |
| MTA2-F-Primer | ATGGCGGCCAACATGTACC |
| MTA2-N-201-R-Primer | CGGAATTCTCCCACAGCTCGGGCCA |
| MTA2-N-312-R-Primer | ggttttccacatgtaataa |
| MTA2-N-462-R-Primer | GGTCAGCTTTGTGGTCTGAA |
| MTA2-N-497-R-Primer | AATGGAGCACTCTGCTTTGAT |
| MTA2-N-542-R-Primer | GATCGGTGTCTTGGTACCCC |
| MTA2-N-624-R-Primer | TTCCAGATGGGTCAGAGCCT |
| MTA2-N-643-R-Primer | AATCAGCGTTGGCTTCACCTTC |
| MTA2-N-664-R-Primer | AATAGGCTCATTGGTGCTGG |
| MTA2-T462G-F-Primer | GGACGTCTTGCCAGACGCATGT |
| MTA2-T462G-R-Primer | CAGCTTTGTGGTCTGAAGCAGG |
| MTA2-I486G-F-Primer | GGAAATGCCAATGCCATCAAA |
| MTA2-I486G-R-Primer | AGGAGCATAAGGCCGTCGG |
| MTA2-I497G-F-Primer | GGACGACTTCCTAAGGCCGCCAAGA |
| MTA2-I497G-R-Primer | GGAGCACTCTGCTTTGATGGCATTG |
| MTA2-T519G-F-Primer | GGAATCGTCAAAGATCTGGTGGCCC |
| MTA2-T519G-R-Primer | TGCCAGGGGCAGCCGCACCAGAGGG |
| MTA2-542-MUG-F-Primer | GGAATCAACAGAAACCAGCTGT |
| MTA2-542-MUG-R-Primer | TGTCTTGGTACCCCGAGGTGT |
| MTA2-I583G-F-Primer | GGACGTTCAAGCTCACAGCCAGCAG |
| MTA2-I583G-R-Primer | CCCTGAAGCCAGAGGCCTTCCATTG |
| MTA2-T621G-F-Primer | GGACATCTGGAAATGCGGCGAGCTG |
| MTA2-T621G-R-Primer | CAGAGCCTTCCGTAGGGCCCTGGTA |
|  | |
| Primers used for qRT-PCR | Sequence (without restriction sites) |
| β-Actin-qRT-F-Primer | ACCGAGCGCGGCTACAG |
| β-Actin-qRT-R-Primer | CTTAATGTCACGCACGATTTCC |
| MTA1-qRT-F-Primer | CCAACATGTACAGGGTCGGA |
| MTA1-qRT-R-Primer | GGTCCGGCCTTATAGCAGAC |
| MTA2-qRT-F-Primer | GGCATTATGGTGAAACGGGC |
| MTA2-qRT-R-Primer | GGGCATCAGCTGGGTTTAGT |
| Elafin-qRT-F-Primer | CACTGTCAAAGGCCGTGTTC |
| Elafin-qRT-R-Primer | GCGGTTAGGGGGATTCAACA |
| NE-qRT-F-Primer | GGAGCCCATAACCTCTCGC |
| NE-qRT-R-Primer | GAGCAAGTTTACGGGGTCGT |
| MTA2-HA-qRT-F-Primer | CCCCAATCCTGTGGTGTTTG |
| MTA2-HA-qRT-R-Primer | AAGCGTAATCTGGAACATCGTA |
| α1-PI-qRT-F-Primer | GTACCCTCAACCAGCCAGAC |
| α1-PI-qRT-R-Primer | TGTCCCCGAAGTTGACAGTG |
| MNEI-qRT-F-Primer | TTCCTGGCGTTGAGTGAGAAC |
| MNEI-qRT-R-Primer | CTGCCGTGTTACCTCTGGTC |
| PI9-qRT-F-Primer | AATGCAAGTGGTACTTTTGCCA |
| PI9-qRT-R-Primer | AAGCCCGATGAATGTCTTCCT |
| SLPI-qRT-F-Primer | CAAACCCAACAAGGAGGAAGC |
| SLPI-qRT-R-Primer | ACAGGGGAAACGCAGGATT |
| Serpin B5-qRT-F-Primer | AATTCGGCTTTTGCCGTTGAT |
| Serpin B5-qRT-R-Primer | TGTCACCTTTAGCACCCACTT |
| Serpin B6-qRT-F-Primer | TGGCACCTTTGCCTTAAACC |
| Serpin B6-qRT-R-Primer | TTCCCTTTGCCCCCATGTAG |
| Serpin B7-qRT-F-Primer | ACACTGCCTCAGGATATGGAA |
| Serpin B7-qRT-R-Primer | TCGGCACACTCAATGTAGTCC |
